# Calcium Signaling Consequences of RyR2-S4938F Mutation Expressed in Human iPSC-Derived Cardiomyocytes

**DOI:** 10.3390/ijms242015307

**Published:** 2023-10-18

**Authors:** Noemi Toth, Xiao-Hua Zhang, Alexandra Zamaro, Martin Morad

**Affiliations:** 1 Cardiac Signaling Center, University of South Carolina, Medical University of South Carolina and Clemson University, Charleston, SC 29425, USA; tothn@musc.edu (N.T.); zhaxi@musc.edu (X.-H.Z.);; 2 Department of Regenerative Medicine and Cell Biology, Medical University of South Carolina, Charleston, SC 29425, USA

**Keywords:** S4938F mutation, RyR2 Ca^2+^ signaling, CRISPR/Cas9, CICR, Ca^2+^ spark, Ca^2+^ release deficiency, hiPSC-CMs

## Abstract

Type-2 ryanodine receptor (RyR2) is the major Ca^2+^ release channel of the cardiac sarcoplasmic reticulum (SR) that controls the rhythm and strength of the heartbeat, but its malfunction may generate severe arrhythmia leading to sudden cardiac death or heart failure. S4938F-RyR2 mutation in the carboxyl-terminal was expressed in human induced pluripotent stem cells derived cardiomyocytes (hiPSC-CMs) using CRISPR/Cas9 gene-editing technique. Ca^2+^ signaling and electrophysiological properties of beating cardiomyocytes carrying the mutation were studied using total internal reflection fluorescence microscopy (TIRF) and patch clamp technique. In mutant cells, L-type Ca^2+^ currents (I_Ca_), measured either by depolarizations to zero mV or repolarizations from +100 mV to –50 mV, and their activated Ca^2+^ transients were significantly smaller, despite their larger caffeine-triggered Ca^2+^ release signals compared to wild type (WT) cells, suggesting I_Ca_-induced Ca^2+^ release (CICR) was compromised. The larger SR Ca^2+^ content of S4938F-RyR2 cells may underlie the higher frequency of spontaneously occurring Ca^2+^ sparks and Ca^2+^ transients and their arrhythmogenic phenotype.

## 1. Introduction

Cardiac excitation–contraction (EC) coupling is the key regulator of cardiomyocyte function as it links the action potential to the contraction. EC coupling is initiated by Ca^2+^ influx through the voltage-gated L-type Ca^2+^ channels (Cav1.2) [1,2], which activate the opening of type-2 ryanodine receptors (RyR2s) of dyadic Ca^2+^ stores of the sarcoplasmic reticulum (SR), triggering a much larger release of Ca^2+^ via the Ca^2+^-induced Ca^2+^ release (CICR) mechanism [1,2,3]. Dyadic releases of Ca^2+^ from the RyR2 appear as localized Ca^2+^ sparks [4]. Sparks last ~7 ms, carrying ~100,000 Ca^2+^ ions that invade the myocyte along its t-tubular system as Ca^2+^ stripes that fully activate the myocyte [4]. The magnitude of the Ca^2+^ current determines the frequency of the Ca^2+^ sparks but not their magnitude [4]. Spatial and temporal summation of the released Ca^2+^ produces an average global rise in cytosolic Ca^2+^ of ~500 nM to ~1000 nM, activating myofilament contraction. Relaxation begins by sequestration of entered and released Ca^2+^ mostly by Ca^2+^ ATPase (SERCA2a) of SR and extrusion of Ca^2+^ across the membrane by Na^+^/Ca^2+^ exchanger (NCX). Since calcium cycling is fundamentally a precise release and uptake process, its dysfunction often underlies cardiac arrhythmias leading to cardiac hypertrophy and failure [5]. In this precise cellular calcium cycling process, the opening and closing of RyR2 play a central role in the activation of calcium transients that regulates the release of Ca^2+^ for cardiac pacing and contraction [6]. Over 150 point mutations in the human type-2 ryanodine receptor gene (*RYR2*) have been identified to associate with catecholaminergic polymorphic ventricular tachycardia (CPVT1). Most of the CPVT1-associated RyR2 mutations cause enhanced Ca^2+^ release with delayed afterdepolarizations (DADs) and arrhythmias and are considered as gain-of-function mutations. The functional consequences of CPVT1-associated RyR2 mutations have been studied mostly in human embryonic kidney (HEK) 293 cell lines expressing recombinant mutant RyR2s and more recently in human iPSC-derived cardiomyocytes (hiPSC-CMs) created directly from patients carrying the mutation or genetically engineered by genome editing [7,8,9,10]. Such studies have shown that CPVT1 may result from SR Ca^2+^ overload- or enhanced RyR2 leakiness-induced aberrant Ca^2+^ releases. Although CPVT1 was the first arrhythmia syndrome linked to gain-of-function of RyR2 [5], more recent studies have identified other inherited arrhythmia syndromes associated with loss of RyR2 function [11,12,13,14,15,16]. The loss-of-function RyR2 mutations and a novel, emerging disease entity termed Ca^2+^ release deficiency syndrome (CRDS) [15,16], appear to be expressed predominantly in the carboxyl-terminal (C-terminal) region. CRDS patients are susceptible to arrhythmogenic events, such as polymorphic ventricular tachycardia, cardiac alternans and ventricular fibrillation, which may result in sudden death. Although several RyR2 mutations, including S4938F RyR2, have been identified as loss-of-function mutations, with a range of clinical phenotypes, there are only few studies reporting the functional consequences of these mutations in human or mammalian cardiomyocyte platforms [17,18,19].

The main regulator of RyR2 is the cytoplasmic calcium, that binds to Ca^2+^ binding residues of RyR2 located at the interface between the central and C-terminal domains. Near-atomic structural analysis of RyR has identified the Ca^2+^-binding sites of RyR2 to be formed essentially by five amino acid residues (E3848, E3922, T4931, H3850 and Q3925), and the caffeine binding site to be located parallel to aromatic chain of W4645, forming a hydrophobic interaction with I4926, and possibly with hydrogen bonds of E4194, suggesting possible allosteric interaction between caffeine and calcium binding sites of RyR2 [20,21,22]. Caffeine is the most used RyR agonist that fully activates RyR2 by sensitizing it to resting calcium, allowing quantification of SR Ca^2+^ content. It has been proposed that CPVT1 or ventricular tachycardia (VT)-associated RyR2 mutations similarly increase the sensitivity of the channel to luminal Ca^2+^, which results in an increased open probability of the channel causing aberrant releases of calcium [23]. It should be noted, however, that near-atomic structural studies of RyR2 show that most CPVT1-associated mutations are not in the Ca^2+^- or caffeine-binding sites, even though they alter the caffeine responses, suggesting that such regulation is mediated through allosteric modulation of the channel. Ca^2+^ release deficiency-associated C-terminal domain mutations have been shown to have varied effects on caffeine activation of RyR2 expressed in HEK293 cells [24], which requires further studies. Here we chose to examine the effects of S4938F-RyR2 mutation located in the C-terminal region, in close proximity to both RyR2 Ca^2+^- and caffeine-binding sites of RyR2 structure [20]. Clinically, S4938F has been identified to cause premature ventricular contractions with a short (<300 ms) coupling interval causing episodic ventricular tachycardia in patients [25]. Studies in HEK293 cells expressing heterologous recombinant S4938F mutant RyR2s have shown lower activity of RyR2 and reduced Ca^2+^ release [25].

In our study, we introduced S4938F-RyR2 mutation in hiPSCs using CRISPR/Cas9 gene editing and characterized the Ca^2+^ signaling properties of S4938F-RyR2 mutant cardiomyocytes. We found higher frequencies of Ca^2+^ sparks and spontaneously triggered Ca^2+^ transients consistent with a larger SR Ca^2+^ content and lower SR Ca^2+^ leak but suppressed magnitude of I_Ca_-gated or spontaneously triggered Ca^2+^ transients.

## 2. Results

### 2.1. Creation of hiPSC Line Harboring the S4938F-RyR2 Mutation

**Figure 1:**
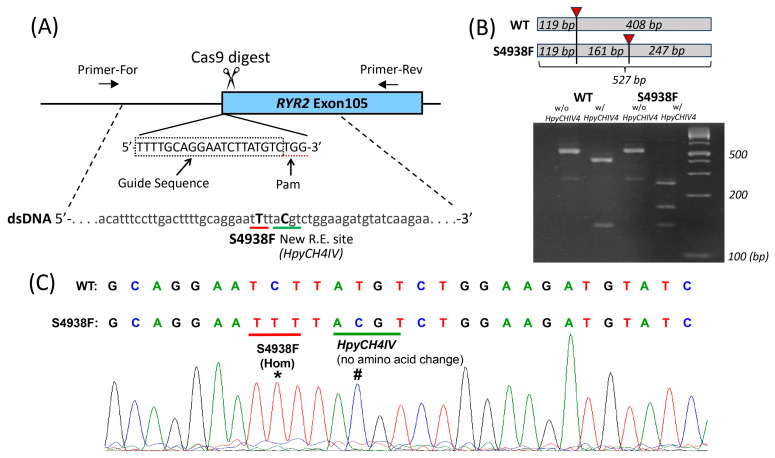
Introduction of S4938F mutation into the ryanodine receptor 2 (*RYR2*) gene of hiPSC-CMs. (**A**) Schematic of gene editing showing *RYR2* gene was digested by Cas9 exonuclease at the target sequence. (**B**) The targeted locus of the genome was amplified by polymerase chain reaction (PCR) with primers F6 and R4 (see Section 4.2), followed by restriction digestion by *HpyCH4IV*. Total size of unrestricted PCR was 527 bp. WT PCR product was cut into two fragments by the enzyme indicated with red triangle (119 + 408 bp). The larger fragment was cut into two fragments in the PCR product of homozygous S4938F mutant, resulting in total of three digested fragments (119 + 161 + 247 bp). (**C**) Sequencing of PCR products show S4938F (TCT to TTT) mutation (*) and a silent mutation creating a new *HpyCHIV4* restriction enzyme site (#).

To introduce the S4938F mutation in the *RYR2* gene of wild type hiPSC-CMs, CRISPR/Cas9 gene-editing technique was used, as described in Section 4.2. (Figure 1A). Mutations were introduced during homology-directed repair with double-stranded plasmid DNA containing a homologous *RYR2* sequence with mutations (Figure 1A). After electroporation of two plasmid DNAs for homology-directed repair and expression of RNA-guided Cas9, cells were screened by puromycin resistance, and each surviving colony was isolated. The DNA of the gene-edited locus of each colony was amplified by PCR to verify correct gene editing. The homologous sequence harbors the S4938F mutation (TCT to TTT) and a silent mutation that creates a new restriction enzyme site *HpyCH4IV*, allowing us to identify the correctly gene-edited clones by restriction enzyme digestion of the PCR products (Figure 1B). Figure 1B shows non-digested and *HpyCH4IV*-digested PCR products of WT and S4938F clones. The full-size PCR product is 527 base pairs (bp) long (Figure 1B). *HpyCH4IV* cuts the PCR product of WT into 408 + 119 bp fragments, while in the homozygous (Hom) S4938F mutant, the 408 bp fragment was further digested into two fragments, resulting in three fragments of 247 + 161 + 119 bp (Figure 1B). Sequencing of PCR products (Figure 1C) showed single peaks of the S4938F mutation (labeled with *) and the silent mutation creating a new *HpyCHIV4* restriction enzyme site (#), confirming that mutations were introduced in both gene alleles, i.e., homozygous mutations.

### 2.2. Spontaneous Ca^2+^ Release in Intact WT and S4938F-RyR2 Mutant hiPSC-CMs

**Figure 2:**
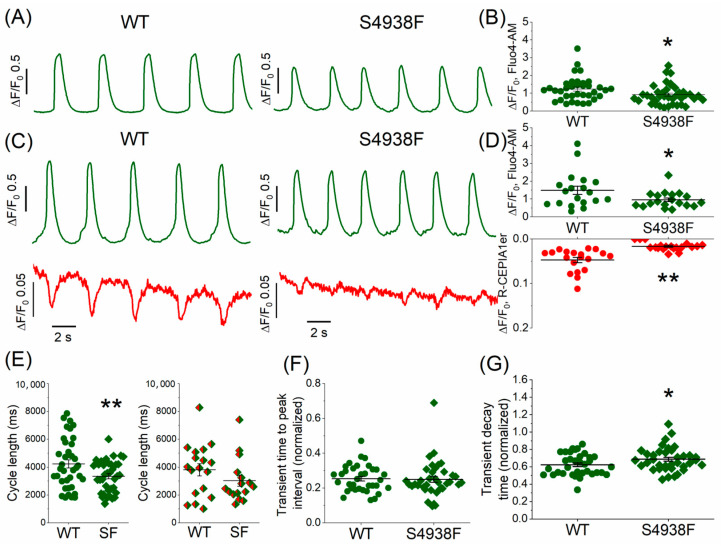
Spontaneous cytoplasmic and SR Ca^2+^ oscillations in intact WT and S4938F-RyR2 mutant hiPSC-CMs. (**A**) Representative traces of spontaneous Ca^2+^ transients measured in WT and S4938F mutant hiPSC-CMs incubated with Fluo-4 AM showing increased spontaneous frequency and decreased Ca^2+^ transients in S4938F. (**B**) Quantified data show that S4938F-RyR2 mutant hiPSC-CMs exhibited decreased spontaneous Ca^2+^ transients as compared with WT hiPSC-CMs (0.92 ± 0.09 ΔF/F_0_ in S4938F vs. 1.23 ± 0.11 ΔF/F0 in WT, n = 39–36, *p* = 0.027). (**C**) Representative traces of spontaneous SR Ca^2+^ releases and accompanying Ca^2+^ transients in WT and S4938F hiPSC-CMs. SR Ca^2+^ release was detected by R-CEPIA1-ER (red) and the cytosolic Ca^2+^ levels were monitored using Fluo-4 AM (green). (**D**) Quantification of spontaneous SR Ca^2+^ release and cytosolic Ca^2+^ changes show greatly reduced SR Ca^2+^ release in the mutant cells (0.02 ± 0.002 ΔF/F_0_ in S4938F vs. 0.05 ± 0.006 ΔF/F_0_ in WT, n = 19–19, *p* = 1.39 × 10^−5^) with suppressed cytosolic Ca^2+^ rise compared with those of WT hiPSC-CMS (0.95 ± 0.10 ΔF/F_0_ in S4938F vs. 1.48 ± 0.23 ΔF/F_0_ in WT, n = 19–19, *p* = 0.04). (**E**) Spontaneous Ca^2+^ transients of Fluo-4 AM loaded mutant cells showed increased frequency (cycle length: 3343.3 ± 186.5 ms in S4938F vs. 4237.7 ± 289.8 ms in WT, n = 37–38, *p* = 0.01, left panel), and the cycle length continued to be shorter in mutant cells loaded with Fluo-4AM along with SR Ca^2+^ probe R-CEPIA1er, although due to a greater variability, the difference was not statistically significant (3026.3 ± 399.2 ms in S4938F vs. 3813.1 ± 444.5 ms in WT, n = 16–18, *p* = 0.20, right panel). (**F**,**G**) Scatter dots show the time-to-peak (TTP) intervals and decay rates of Ca^2+^ transients measured by Fluo-4 AM in WT and S4938F mutant hiPSC-CMs. TTP and decay times of Ca^2+^ transients were quantified as a ratio normalized to their cycle lengths, showing unaltered TTP (0.25 ± 0.02 in S4938F vs. 0.25 ± 0.01 in WT, n = 37–35, *p* = 0.84) and slower decay in S4938F mutant hiPSC-CMs (0.69 ± 0.02 in S4938F vs. 0.62 ± 0.02 in WT, n = 37–35, *p* = 0.04). Data are shown as scatter dots with mean ± SEM. * *p* < 0.05, ** *p* < 0.01.

Unlike adult ventricular cardiomyocytes, WT hiPSC-CMs exhibit spontaneous automaticity. To determine whether the spontaneous Ca^2+^ release characteristics of S4938F RyR2 mutant cells were altered compared with WT cells, in one set of experiments we measured the cytosolic Fluo-4 AM Ca^2+^ transients (Figure 2A,B), and in another set of experiments we also measured their Ca^2+^ release signal simultaneously using the SR-targeted Ca^2+^ probe R-CEPIA1er along with their cytosolic Ca^2+^ transients (Figure 2C,D). Figure 2A,E (left panel) show that mutant cells loaded with Fluo-4 AM had faster spontaneous oscillation frequency compared to WT (cycle length 3343.3 ± 186.5 ms in S4938F vs. 4237.7 ± 289.8 ms in WT). Similarly, the cycle length seemed to be shorter in S4938F when the cells were infected with the SR-targeted probe R-CEPIA1er along with Fluo-4 AM, although the difference was not statistically significant (3026.3 ± 399.2 ms in S4938F vs. 3813.1 ± 444.5 ms in WT, Figure 2E (right panel)). While spontaneously beating WT hiPSC-CMs showed both significant cytosolic Ca^2+^ transients (Fluo-4 AM) and SR Ca^2+^ release, S4938F cells failed to generate significant SR Ca^2+^ release signals (0.02 ± 0.002 ΔF/F_0_ in S4938F vs. 0.05 ± 0.006 ΔF/F_0_ in WT, Figure 2C,D). Note that the amplitude of spontaneous cytosolic Ca^2+^ transients (Fluo4-AM signal) was also significantly suppressed in S4938F mutant hiPSC-CMs, both measured alone (0.92 ± 0.09 ΔF/F_0_ in S4938F vs. 1.23 ± 0.11 ΔF/F0 in WT, Figure 2A,B) or along with the SR Ca^2+^ sensor (0.95 ± 0.10 ΔF/F_0_ in S4938F vs. 1.48 ± 0.23 ΔF/F_0_ in WT, Figure 2C,D).

The time-to-peak intervals of spontaneous Ca^2+^ transients were essentially the same between WT and S4938F cells (normalized to their cycle length: 0.25 ± 0.02 in S4938F vs. 0.25 ± 0.01 in WT, Figure 2F). The decay time of Ca^2+^ transients in S4938F cells was longer than in WT hiPSC-CMs when normalized to their cycle length: 0.69 ± 0.02 in S4938F vs. 0.62 ± 0.02 in WT, Figure 2G). The longer decay of Ca^2+^ transients raise the possibility of slower inactivation of I_Ca_.

### 2.3. SR Ca^2+^ Content of WT and S4938F-RyR2 Mutant hiPSC-CMs

**Figure 3:**
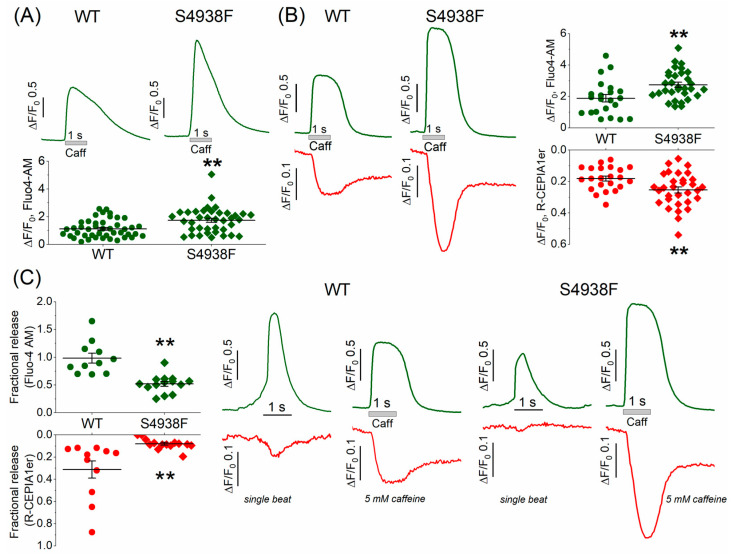
Sarcoplasmic reticulum Ca^2+^ content measured by 5 mM caffeine. (**A**) Representative traces and quantified data of 5 mM caffeine-triggered Ca^2+^ transients measured by Fluo-4 AM in intact WT and S4938F mutant hiPSC-CMs. Caffeine-induced Ca^2+^ transients were significantly higher in S4938F cells (1.73 ± 0.15 ΔF/F_0_ vs. 1.12 ± 0.1 ΔF/F_0_, n = 38–44, *p* = 0.0007). (**B**) Simultaneously measured caffeine-triggered cytosolic Ca^2+^ transient (green) and SR Ca^2+^ release (red) also showed higher Ca^2+^ release (0.25 ± 0.019 ΔF/F_0_ vs. 0.18 ± 0.016 ΔF/F_0_, n = 31–22, *p* = 0.009) and cytosolic Ca^2+^ rise in S4938F cells compared with those of WT cells (2.74 ± 0.16 ΔF/F_0_ vs. 1.88 ± 0.24 ΔF/F_0_, n = 31–22, *p* = 0.003). (**C**) Quantified data and representative traces show that fractional release of Ca^2+^ as the ratio of spontaneous Ca^2+^ release to caffeine-induced Ca^2+^ release was significantly smaller in S4938F mutant cells (R-CEPIA1er: 0.08 ± 0.01 vs. 0.31 ± 0.08, n = 14–11, *p* = 0.003; Fluo-4 AM: 0.52 ± 0.04 vs. 0.98 ± 0.09, n = 14–11, *p* = 4.6 × 10^−5^). Data are shown as scatter plots with mean ± SEM, ** <0.01.

SR Ca^2+^ content was measured by rapid application of 5 mM caffeine in intact WT and S4938F mutant hiPSC-CMs. In Fluo-4 AM loaded mutant cells, caffeine-induced cytosolic Ca^2+^ rise was significantly higher than WT cells (1.73 ± 0.15 ΔF/F_0_ vs. 1.12 ± 0.1 ΔF/F_0_, Figure 3A). In mutant cells, where SR Ca^2+^ release signal and cytosolic Ca^2+^ transients were measured simultaneously, 5 mM caffeine also consistently caused significantly larger SR Ca^2+^ release signal (0.25 ± 0.019 ΔF/F_0_ vs. 0.18 ± 0.016 ΔF/F_0_) and cytosolic Ca^2+^ rise compared to WT cells (2.74 ± 0.16 ΔF/F_0_ vs. 1.88 ± 0.24 ΔF/F_0_) (Figure 3B). Fractional Ca^2+^ release, defined as the ratio of spontaneous Ca^2+^ release to caffeine-triggered Ca^2+^ release, was larger in WT compared to S4938F cells (R-CEPIA1er: 0.08 ± 0.01 in S4938F vs. 0.31 ± 0.08 in WT; Fluo-4 AM: 0.52 ± 0.04 in S4938F vs. 0.98 ± 0.09 in WT, Figure 3C). The larger caffeine-triggered cytoplasmic Ca^2+^ rise in the mutant cells indicated higher SR Ca^2+^ levels, but the smaller spontaneous Ca^2+^ release transients suggest impaired I_Ca_ gating and down-regulated CICR.

**Figure 4:**
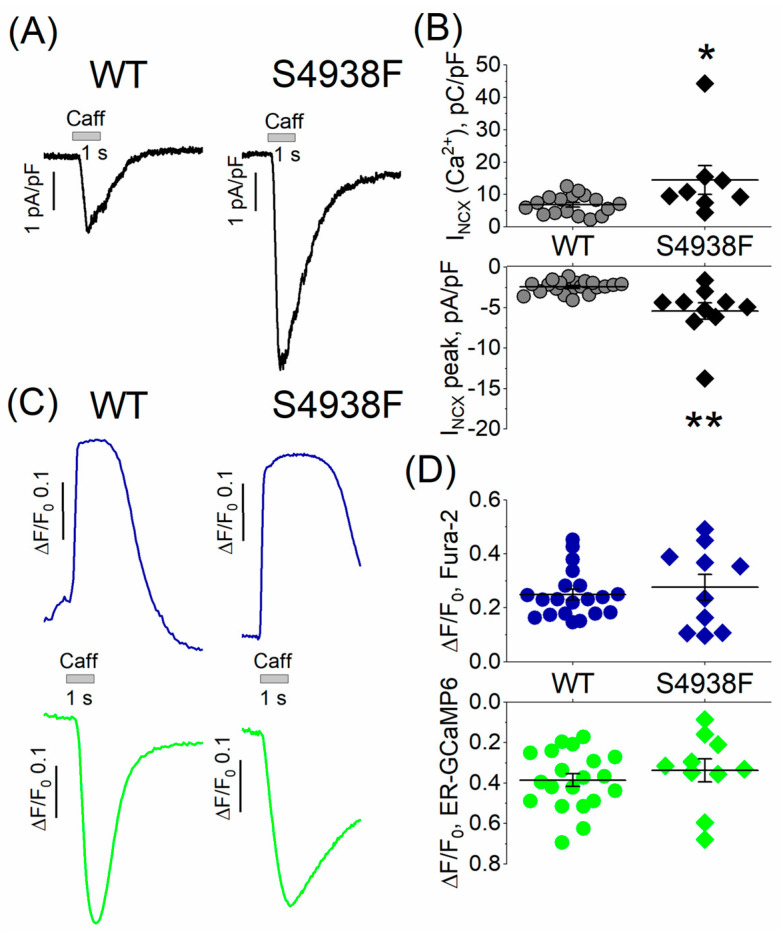
I_NCX_ and caffeine-induced Ca^2+^ release from patch-clamped and TIRF-imaged WT and S4938F mutant hiPSC-CMs. (**A**) Representative traces of I_NCX_ from WT and S4938F mutant cells. (**B**) Quantified I_NCX_ densities from WT and mutant cells. Scatter plots show that both peak I_NCX_ (5.42 ± 1.03 pA/pF vs. 2.45 ± 0.2 pA/pF, n = 10–20, *p* = 0.0005) and the carried Ca^2+^ by I_NCX_ (14.5 ± 4.4 pC/pF vs. 6.8 ± 0.8 pC/pF, n = 8–17, *p* = 0.02) were significantly higher in S4938F mutant hiPS-CMs compared with those of WT cells in 5 mM extracellular calcium. (**C**) Representative traces of caffeine-induced SR Ca^2+^ release (ER-GCaMP6, green) and cytosolic Ca^2+^ rise (Fura-2, blue) in WT and S4938F mutant CMs. (**D**) Scatter plots depict no significant difference in the caffeine-induced Ca^2+^ release or Ca^2+^ transients in patched conditions compared with WT (0.34 ± 0.06 ΔF/F_0_ in S4938F vs. 0.39 ± 0.03 ΔF/F_0_ in WT, n = 10–20, *p* = 0.4, and 0.27 ± 0.05 ΔF/F_0_ in S4938F vs. 0.26 ± 0.02 ΔF/F_0_ in WT, n = 10–20, *p* = 0.7, respectively). Data are shown as scatter plots with mean ± SEM. * *p* < 0.05, ** *p* < 0.01.

In whole cell patch-clamped and TIRF-imaged cells, caffeine-activated peak I_NCX_ was also significantly larger in mutant compared with WT cells (5.4 ± 1.03 pA/pF vs. 2.5 ± 0.2 pA/pF, Figure 4A,B), consistent with the enhanced caffeine-triggered Ca^2+^ stores in mutant cells (Figure 3A,B). The net charge carried by I_NCX_ was also significantly larger in S4938F hiPSC-CMs compared with WT (14.5 ± 4.4 pC/pF vs. 7.2 ± 0.8 pC/pF, Figure 4A,B). Despite increased I_NCX_ in S4938F mutant hiPSC-CMs, there was no difference in the caffeine-induced SR Ca^2+^ release or cytosolic Ca^2+^ transients in patched conditions compared with WT (0.34 ± 0.06 ΔF/F_0_ in S4938F vs. 0.39 ± 0.04 ΔF/F_0_ in WT and 0.28 ± 0.05 ΔF/F_0_ in S4938F vs. 0.26 ± 0.02 ΔF/F_0_, respectively, Figure 4C,D). This discrepancy between the calcium indicator probes and membrane current measurements of calcium release signal simultaneously measured in patch-clamped myocytes may result from fluorescent probe-calcium saturation kinetics or cellular calcium buffering consequences of whole cell patched cells. Nevertheless, I_NCX_ measurements of enhanced cytosolic calcium release in voltage-clamped mutant cells (Figure 4) are consistent with enhanced fluorescent probe signal measurements of calcium in intact non-patched mutant cells of Figure 3.

### 2.4. I_Ca_ and I_Ca_-Induced Ca^2+^ Release in WT and S4938F-RyR2 hiPSC-CMs

**Figure 5:**
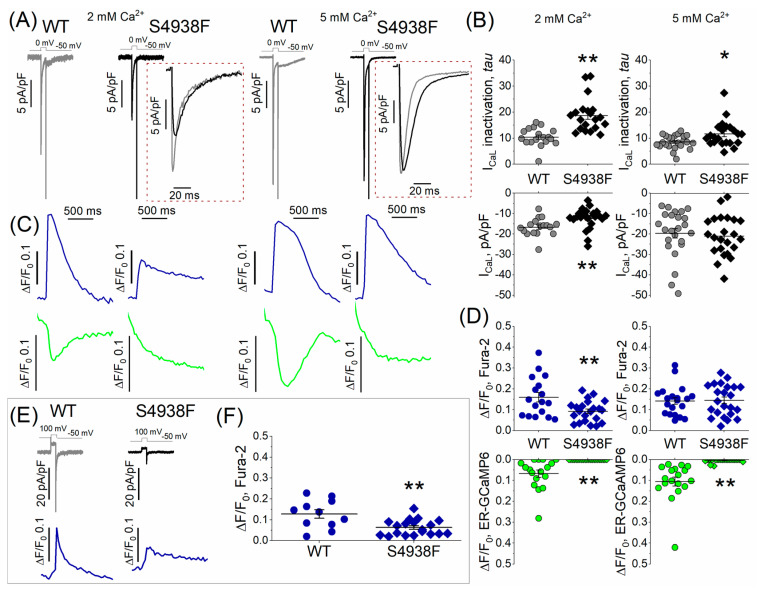
L-type Ca^2+^ current (I_Ca_) and Ca^2+^-induced Ca^2+^ release (CICR). (**A**) Representative traces of I_Ca_ recorded on depolarizations to zero mV from holding potential of –50 mV in WT and S4938F mutant hiPSC-CMs. (**B**) Quantified I_Ca_ density (bottom panel) and I_Ca_ inactivation (top panel) from WT and S4938F-RyR2 mutant hiPSC-CMs. I_Ca_ density was significantly smaller in S4938F hiPSC-CMs in 2 mM extracellular Ca^2+^ solutions (12.35 ± 1.1 pA/pF vs. 16.62 ± 1.05 pA/pF, n = 22–17, *p* = 0.009), and the current inactivated significantly slower (inactivation *tau*: 18.74 ± 1.45 ms in S4938F vs. 10.41 ± 0.82 ms in WT, n = 20–17, *p* = 2.3 × 10^−5^). Increasing the extracellular Ca^2+^ concentration from 2 mM to 5 mM, increased the I_Ca_ density in both cell types (21.0 ± 2.05 pA/pF in S4938F vs. 19.6 ± 2.4 pA/pF in WT, n = 23–24, *p* = 0.66). (**C**) Representative traces of I_Ca_-activated Ca^2+^ transients and SR Ca^2+^ release in WT and S4938F hiPSC-CMs measured by Fura2 pentasodium salt (blue) and SR targeted probe (ER-GCaMP6, green). (**D**) CICR was suppressed in S4938F hiPSC-CMs compared with levels in WT hiPSC-CMs (0 ΔF/F_0_ in S4938F vs. 0.07 ± 0.02 ΔF/F_0_ in WT, n = 22–17, *p* = 0.0001). Increasing the extracellular Ca^2+^ concentration from 2 mM to 5 Mm elevated the cytosolic Fura-2 Ca^2+^ signals in mutant cells to levels comparable with those of WT cells (0.15 ± 0.02 ΔF/F_0_ in S4938F vs. 0.14 ± 0.02 in WT, but did not significantly enhance I_Ca_-induced Ca^2+^ release signal from the SR. (**E**) I_Ca_ tail current and I_Ca_ tail current triggered Ca^2+^ transients in WT and S4938F cells. (**F**) Quantified data of I_Ca_ tail current-induced fluorescence signals indicate significantly smaller Ca^2+^ transients in S4938F cells in 2 mM extracellular Ca^2+^ (0.06 ± 0.009 ΔF/F_0_ in S4938F vs. 0.13 ± 0.02 ΔF/F_0_ in WT, n = 19–11, *p* = 0.002). Data are shown as scatter plots with mean ± SEM. * *p* < 0.05, ** *p* < 0.01.

I_Ca_ and activation of SR Ca^2+^ release and the resultant cytosolic Ca^2+^ transients were measured in patch-clamped and TIRF-imaged WT and S4938F hiPSC-CMs dialyzed with 0.1 mM Fura-2-pentasodium salt through the patch pipette. To minimize the contribution of the influx of calcium during the 100 ms long depolarization pulse, yet to activate fully the channel, I_Ca_ tail current-induced Ca^2+^ transients were measured using repolarization pulses from 100 mV to −50 mV. Figure 5A and C show representative recordings of I_Ca_ and the accompanying SR Ca^2+^ release signals and cytosolic Ca^2+^ transients measured by ER-GCaMP6 and Fura-2, respectively, while Figure 5E shows I_Ca_ tail current-induced Ca^2+^ transients measured by Fura-2. I_Ca_ density was significantly smaller in S4938F hiPSC-CMs in 2 mM extracellular Ca^2+^ solutions (12.35 ± 1.1 pA/pF vs. 16.62 ± 1.05 pA/pF), and the current inactivated significantly slower (Figure 5A,B). I_Ca_ inactivation in S4938F hiPSC-CMswas significantly slower as compared to WT hiPSC-CMs, measured either in 2 or 5 mM extracellular Ca^2+^ (18.74 ± 1.45 ms in S4938F vs. 10.41 ± 0.82 ms in WT, and 11.63 ± 1.04 ms in S4938F vs. 8.57 ± 0.52 ms in WT), respectively (Figure 5A,B (insets)).

I_Ca_ activated significantly suppressed cytosolic Ca^2+^ transients in S4938F mutant hiPSC-CMs (0.093 ± 0.01 ΔF/F_0_ in S4938F vs. 0.16 ± 0.02 ΔF/F_0_ in WT), even when normalized to the smaller current density, and failed to trigger SR Ca^2+^ release compared with WT cells (0 ΔF/F_0_ in S4938F vs. 0.07 ± 0.02 ΔF/F_0_ in WT, Figure 5C,D). As the Ca^2+^ release was completely attenuated in 2 mM extracellular Ca^2+^ conditions, we tried to enhance the Ca^2+^ release by increasing the calcium to 5 mM in the extracellular solution. Increasing the extracellular Ca^2+^ concentration from 2 mM to 5 mM, increased the I_Ca_ density in both cell types (21.0 ± 2.05 pA/pF in S4938F vs. 19.6 ± 2.4 pA/pF in WT, Figure 5B (right)) and elevated the cytosolic Fura-2 Ca^2+^ signals in mutant cells to levels comparable with those in WT cells (0.15 ± 0.02 ΔF/F_0_ in S4938F vs. 0.14 ± 0.02 in WT, Figure 5D (right panel)), but did not significantly enhance the I_Ca_-induced Ca^2+^ release signal from the SR, Figure 5D (right, green).

Quantified data of I_Ca_ tail current-induced Ca^2+^ transients indicate significantly reduced cytosolic Ca^2+^ transients in S4938F cells in 2 mM extracellular Ca^2+^ (0.06 ± 0.009 ΔF/F_0_ in S4938F vs. 0.13 ± 0.02 ΔF/F_0_ in WT), Figure 5E,F.

### 2.5. Ca^2+^ Sparks in WT and S4938F-RyR2 Mutant hiPSC-CMs

**Figure 6:**
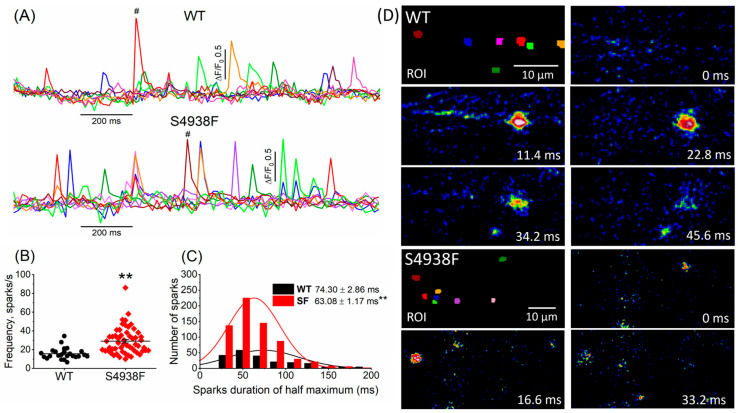
Ca^2+^ sparks recorded from WT and S4938F-RyR2 mutant hiPSC-CMs. (**A**) Representative time courses of normalized Ca^2+^ sparks from different color-coded regions in WT and S4938F mutant cells. (**B**) Scatter plot shows significantly higher spark frequency of S4938F mutant hiPSC-CMs compared with WT cells (29.0 spark/s in S4938F vs. 15.7 spark/s in WT, n = 55–24, *p* = 2.9 × 10^−5^). (**C**) Histogram and distribution curves depict the spark duration measured at half-maximum amplitude (63.08 ± 1.17 ms in S4938F vs. 74.30 ± 2.86 ms in WT, n = 212–662, *p* = 2.17 × 10^−5^). (**D**) Total internal reflection fluorescence images show the evolution of the # labeled sparks from WT and S4938F mutant hiPSC-CMs. Data are shown as scatter plots with mean ± SEM, ** *p* < 0.01.

Ca^2+^ sparks were measured in intact S4938F RyR2 mutant and WT cells incubated with Fluo4-AM. Two-dimensional imaging of spontaneously igniting calcium sparks were recorded from different cellular regions of interest (ROI). Figure 6A shows the time courses of Ca^2+^ sparks recorded from different color-coded cellular regions in WT and S4938F mutant cells. Spontaneous sparks ignited more frequently in S4938F mutants compared with WT hiPSC-CMs (29.0 spark/s in S4938F vs. 15.7 spark/s in WT) (Figure 6B), consistent with the elevated SR Ca^2+^ content. The sparks histogram in Figure 6C depicts the spark duration distribution that averaged 74.30 ± 2.86 ms in WT and 63.08 ± 1.17 ms in S4938F mutant cells. Although spark durations showed larger cellular variability in WT cells, resulting in longer average duration, their peak occurrences in the number of the sparks were similar in both cell lines. Figure 6D shows TIRF images of the evolution of Ca^2+^ sparks (labeled by #) in WT and S4938F mutant cells.

### 2.6. Ca^2+^ Leak in WT and S4938F Mutant hiPSC-CMs

**Figure 7:**
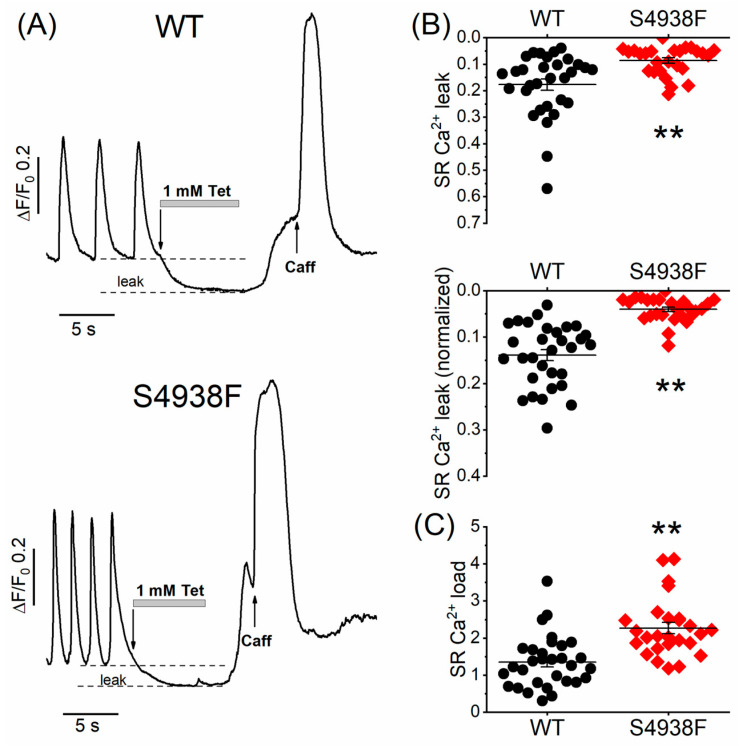
Ca^2+^ leak and load levels in WT and S4938F mutant hiPSC-CMs. (**A**) Representative time courses of normalized Ca^2+^ fluorescence changes in spontaneously beating WT and S4938F mutant hiPSC-CMs after rapid exposure to zero Ca^2+^/Na^+^ solution containing 1 mM tetracaine followed by 5 mM caffeine. (**B**) Scatter plots show decreased raw sarcoplasmic reticulum (SR) Ca^2+^ leak levels (0.09 ± 0.01 ΔF/F_0_ vs. 0.18 ± 0.02 ΔF/F_0_, n = 26–31, *p* = 0.0007) and decreased SR Ca^2+^ levels normalized to the SR Ca^2+^ store size (0.04 ± 0.005 ΔF/F_0_ vs. 0.14 ± 0.012 ΔF/F_0_, n = 26–31, *p* = 2.7 × 10^−9^). (**C**) Along with the reduced SR Ca^2+^ leak, S4938F cells showed higher SR Ca^2+^ load triggered by caffeine (2.27 ± 0.15 ΔF/F_0_ vs. 1.35 ± 0.13 ΔF/F_0_, n = 26–31, *p* = 1.94 × 10^−5^). Data are shown as scatter plots with mean ± SEM, ** *p* < 0.01.

To measure whether S4938F RyR2 mutation altered the magnitude of the SR Ca^2+^ leak, WT and mutant cells were first bathed in a normal Tyrode solution that supports spontaneous Ca^2+^ releases and beating and then cells were exposed to a zero Ca^2+^/Na^+^ solution containing 1 mM tetracaine, followed by rapid application of 5 mM caffeine to quantify the SR Ca^2+^ content. SR Ca^2+^ leak was quantified as the decrease in the diastolic fluorescence level in the zero Ca^2+^/Na^+^ solution and then was normalized to the SR Ca^2+^ content triggered by caffeine. Figure 7A shows representative time courses of normalized Ca^2+^ fluorescence changes in spontaneous beating WT and S4938F cells after application of tetracaine followed by 5 mM caffeine in zero Ca^2+^/Na^+^ solution. Both the raw (0.09 ± 0.01 ΔF/F_0_ vs. 0.18 ± 0.03 ΔF/F_0_, Figure 7B (top)) and normalized SR Ca^2+^ leak (0.04 ± 0.005 ΔF/F_0_ vs. 0.13 ± 0.015 ΔF/F_0_, Figure 7B (bottom)) were significantly smaller in S4938F hiPSC-CMs than those of WT hiPSC-CMs, consistent with the higher SR Ca^2+^ load triggered by 5 mM caffeine (2.27 ± 0.15 ΔF/F_0_ vs. 1.51 ± 0.16 ΔF/F_0_, Figure 7C). Although higher SR Ca^2+^ content should have induced higher Ca^2+^ leak in S4938F cells, we consistently found reduced SR Ca^2+^ leak, supporting the possibility of allosteric modulation of the channel.

## 3. Discussion

The major finding of this study was that I_Ca_-gated or spontaneously triggered calcium release signals of S4938F-RyR2 mutant hiPSC-CMs were suppressed compared with WT myocytes even though the caffeine-triggered SR Ca^2+^ release was larger and the SR Ca^2+^ leak was smaller. It is likely that mutation induces either a conformational change in the channel or the cellular proximity of dyads to the surface membrane is altered, leading to impaired CICR. Structural dissociation of dyads from the plasma membrane may also alter the regulatory control of calcium channels over RyR2, which may lead to the observed enhanced frequency of calcium sparks in mutant S4938F cells. The higher Ca^2+^ spark frequency along with the higher SR Ca^2+^ content is most likely responsible for the arrhythmogenic phenotype of the S4938F mutation.

### 3.1. Enhanced SR Ca^2+^ Content of S4938F-RyR2 Mutant hiPSC-CMs

The SR Ca^2+^ content was measured using the R-CEPIA1er Ca^2+^ sensor targeted to SR and monitoring the Fluo-4 AM cytosolic signal in response to rapid application of 5mM caffeine or by measuring I_NCX_ triggered by caffeine-induced intracellular Ca^2+^ rise. Application of 5 mM caffeine induced significantly larger Ca^2+^ transients and I_NCX_ in S4938F hiPSC-CMs than in WT hiPSC-CMs, consistent with a higher Ca^2+^ content of SR in S4938F-RyR2 mutant hiPSC-CMs. It is likely that the smaller calcium leak of S4938F mutant cells causes the higher Ca^2+^ content of SR, which may underlie the higher frequency of spontaneously occurring Ca^2+^ sparks or Ca^2+^ waves (Figure 6). The higher frequency of occurrence of Ca^2+^ sparks in S4938F mutant hiPSC-CMs is consistent with the higher Ca^2+^ waves frequency reported in HL-1 cardiomyocytes expressing S4938F-RyR2 [26]. The suppressed spontaneous calcium release also contributes to the increased SR Ca^2+^ stores, as in the absence of significant release, the accumulation of the residual Ca^2+^ in the SR increases.

### 3.2. Suppression of Spontaneous and I_Ca_-Gated SR Ca^2+^ Release in S4938F-RyR2 Mutants

A previous study in HEK293 cells expressing recombinant S4938F-RyR2 mutation failed to show spontaneous Ca^2+^ oscillations using Fura-2 AM probe [25,26]. In sharp contrast, our data in the hiPSC-CM platform not only show that the S4938F-RyR2 mutant exhibited spontaneous cytosolic Ca^2+^ oscillations, but also that their frequency of occurrence was significantly higher than those measured in WT cells. It should be noted, however, that the spontaneous SR Ca^2+^ releases were significantly smaller and relaxed slowly. Since the decay times of spontaneous Ca^2+^ transients were longer, an impaired SERCA2a function could not be ruled out in mutant cells. This finding, along with significant suppression of CICR in S4938F compared with WT hiPSC-CMs, is consistent with the studies of Fujii et al. showing greatly reduced [^3^H]ryanodine binding and decreased CICR activity in S4938F mutant HEK293 cells [25]. Since increasing the extracellular Ca^2+^ concentration to 5 mM failed to enhance the SR Ca^2+^ release, we suggest that decreased Ca^2+^ gating of mutant RyR2 underlies the phenotype of this mutation in heart cells.

### 3.3. Pathophysiological Implications of S4938F-RyR2 Mutation

Clinically, S4938F-RyR2 mutation has been identified in patients who have bouts of premature ventricular contractions with a short (<300 ms) coupling interval, causing episodic ventricular tachycardia [25].

We chose to study the homozygous variant of this mutation, even though in patients this mutation is expressed as heterozygous variant, to minimize complications arising from the heterotetrametric structure of RyR2. Although this approach does not fully reflect the patient pathology, it does provide more precise data on the structure function implications of the mutation.

The S4938F site is located in the carboxyl-terminal tail of RyR2 that directly lines the helix of the ion channel conducting pore in close proximity to the channel’s activation core where the channel binding sites for Ca^2+^, caffeine and ATP are also located. It is not surprising, therefore, that several human-disease-associated C-terminal and pore mutations are expressed as “loss-of-function” mutations. It is likely, therefore, that S4938F-RyR2 mutation affects the transmission of the activating ligand signal to the C-terminal that regulates the ion channel pore open/close state. Indeed, Ca^2+^-dependent activation of RyR2 in recombinant protein platforms have shown such reduced activating Ca^2+^ affinity in the S4938F-RyR2 mutant, consistent with our I_Ca_-induced Ca^2+^ release results. It remains of interest to investigate whether “affinities” to caffeine or other activating ligands are similarly altered by S4938F mutation. We have already reported that mutations in the calcium binding site that disable CICR also suppress caffeine-triggered responses, which suggests crossover effects between calcium and other ligand binding site functions [7]. It was, therefore, unexpected to find that in S4938F-expressing cells, even though CICR was suppressed, the caffeine-triggered responses were enhanced, suggesting a more complex interaction between the pore and calcium binding site residues. It is likely that there may be crossover effects of this mutation on other binding sites of the channel, which helps to preserve or enhance the caffeine response even when CICR is suppressed.

## 4. Materials and Methods

### 4.1. Cell Culture of hiPSC Lines

The wild-type (K3) line of hiPSC was established previously by Si-Tayeb et al. [27] and was provided for our studies. The cell line was maintained in StemFlex medium (Gibco, Thermo Fisher Scientific, Waltham, MA, USA) on Vitronectin-coated dishes at 37 °C with 5% CO_2_. The culture medium was replaced every 2 days and hiPSCs were passaged with Accutase (Thermo Fisher) every 4–6 days. A 10 μM quantity of Y-27632 ROCK inhibitor (Tocris) was added to the culture media for 24 h after each passage. Cell passages between No. 50 and No. 60 were used for the experiments.

### 4.2. CRISPR/Cas9 Genome Editing

CRISPR/Cas9 gene editing technique was used to introduce the S4938F mutation into the *RYR2* gene of hiPSCs. The guide sequence 5′-TTTTGCAGGAATCTTATGTC-3′ adjacent to the Pam sequence (TGG) was identified in exon105 of the *RYR2* gene by CRISPR Design Tool and was cloned into the pSpCas9 (BB)-2A-Puro vector (pX459, Addgene) to guide Cas9 exonuclease (Figure 1A), which digests the *RYR2* gene within the guide sequence. Site-directed mutations were introduced by homology-directed repair of the Cas9-digested site using double-stranded plasmid DNA containing a ~400 bp homologous *RYR2* genome sequence with mutations (Figure 1A). The homologous sequence harbors the S4938F mutation (TCT to TTT) and a silent mutation (no amino acid change: TAC to TAT) that creates a new restriction enzyme site *HpyCH4IV* to facilitate a process screening for correctly gene-edited hiPSC clones (Figure 1B,C). The plasmid DNA expressing RNA-guided Cas9 (15 µg) and the linearized double-stranded plasmid DNA (5 µg) carrying mutations were co-transfected by electroporation with two 30 ms pulses at 1050 V. The transfected cells were then plated in 60 mm vitronectin-coated dishes with 10 μM Y-27632 ROCK inhibitor in the culture medium. Then, 24 h after transfection, the cells were incubated with 1 μg/mL puromycin (Thermo) for 48 h. The surviving cell colonies were harvested 7–10 days after antibiotics treatment. Genomic DNAs of each cell colony were extracted from the harvested cells, and the S4938F mutations were screened by polymerase chain reaction (PCR) followed by restriction enzyme *HpyCH4IV* digestion (Figure 1B), and direct sequencing of PCR products (Figure 1C). Two sets of PCR primer pairs, F4xR4 or F6xR4, were used for screening the colonies (sequences shown below). Figure 1C shows the S4938F mutation site (*) and the silent mutation creating a new *HpyCHIV4* restriction enzyme site (#) which was used for screening correct cell clones.

Screening primers for S4938F:

F4-5′-AGG CTG GTC TTG AAT GCC TG-3′

F6-5′-AAC TCA AGC GAT CCT CCC AC-3′

R4-5′-GGA GGA AAC AGC TCT TCC GA-3′.

### 4.3. Differentation of hiPSCs into Cardiomyocytes

Wild type and S4938F mutant hiPSCs were differentiated into cardiomyocytes in 24-well plates, as described in detail previously [28,29]. Briefly, the cells were dissociated in Matrigel (Corning)-coated wells and were treated with 10 µM CHIR 99021, a GSK3β inhibitor, for 24 h in RPMI/B-27 without insulin. Then, 72 h after this treatment, 5 µM IWR-1, a Wnt signaling inhibitor, was added to the cell cultures for 48 h in RPMI/B-27 without insulin. After removal of the drug, the cells were cultured with RPMI/B-27 without insulin until spontaneous beating started. Once beating was observed, the medium was changed, and the cells were kept in RPMI/B-27 with insulin medium.

### 4.4. Dissociation of Single hiPSC-CMs for Electrophysiological Experiments

The beating hiPSC-CMs were grown in monolayer cell cultures for a minimum of 5 weeks before they were enzymatically (TrypLE Select Enzyme 10×, Gibco) dissociated into single cardiomyocytes for electrophysiological and Ca^2+^ signaling experiments. The cells were plated on Vitronectin- or Matrigel-coated 25 mm glass coverslips and were incubated for three to four days before the experiments.

### 4.5. ER Ca^2+^ Probes

ER-GCaMP6-150 cDNA plasmid [30] (#86918) and R-CEPIA1er plasmid [31] (#58216) were purchased from Addgene (Watertown, MA, USA). Adenovirus carrying ER-GCaMP6-150 was produced by Vector Biosystems Inc (Malvern, PA, USA), and adenovirus carrying R-CEPIA1er was produced by Welgen Inc. (Worcester, MA, USA). At 24–48 h after plating the hiPSC-CMs on 25 mm coverslips, the cells were infected by a solution containing ER-GCaMP6 or R-CEPIA1er adenovirus containing (MOI) of 300–500 virus particles per cell (vp/cell). After 6–8 h, the virus medium was removed, and hiPSC-CMs were supplemented with B27+ medium at 37 °C until their use between 48 and 72 h post infection.

### 4.6. Ca^2+^ Imaging

WT and S4938F mutant hiPSC-CMs were imaged using a total internal reflection fluorescence (TIRF) imaging system. Directly before the experiments, the cells were loaded with either 1 µM Fluo-4 AM or 1 µM Fura-2 AM fluorescent dyes for 20 min at 37 °C in Tyrode’s solution containing (in mM) 137 NaCl, 5.4 KCl, 2 CaCl_2_, 1 MgCl_2_, 10 glucose and 10 HEPES, titrated to pH 7.4 with NaOH. Ca^2+^ transient and Ca^2+^ sparks were recorded at 70–80 Hz with a depth of penetration of 150 nm. Image sequences and fluorescence signals were detected and analyzed with LASX Leica software (Leica Microsystems Inc., Wetzlar, Germany). The fluorescence signals were quantified as the ratio of the changes in whole-cell fluorescence (ΔF) and the baseline fluorescence level (F_0_). The Ca^2+^ sparks were exported and analyzed with our custom-developed Con2j software [32]. The method of Ca^2+^ spark analysis was illustrated and described previously [4,32].

### 4.7. SR Ca^2+^ Leak and Load Measurement

Spontaneously beating WT and mutant hiPSC-CMs were imaged after 25–30 min of incubation of Fluo-4 AM at 37 °C. The Ca^2+^ leak of the SR was measured in spontaneously contracting cells when normal Tyrode solution was rapidly switched to zero Na^+^/Ca^2+^ solution containing 1 mM tetracaine to block RyR2. After tetracaine removal, the SR Ca^2+^ load was measured by rapid administration of 5 mM caffeine. The SR Ca^2+^ leak was quantified as the difference between the baseline fluorescence level and tetracaine-induced decrease in the diastolic Ca^2+^ level.

### 4.8. Electrophysiological Measurements

WT and S4938F mutant hiPSC-CMs were voltage-clamped in the whole cell configuration mode of patch clamp technique. The membrane voltage was obtained using a Dagan amplifier connected to a Digidata 1320A (Molecular Devices, San Jose, CA, USA) analogue–digital converter and pClamp software (Clampex 10.6, Molecular Devices). Borosilicate patch pipettes with resistances of 2–4 MΩ were pulled using a horizontal pipette puller (Model P-87, Sutter Instruments, Novato, CA, USA). The cells were dialyzed with Cs^+^-based internal solution containing (in mM) 100 Cs^+^-Aspartate, 15 NaCl, 30 TEA-Cl, 5 Mg-ATP, 0.5 MgCl_2_, 0.2 EGTA, 0.3 CaCl_2_, 10 HEPES and 0.1 Fura-2 pentasodium salt, at pH 7.2 and an osmolality of 300 Osmls. I_Ca_ was activated by a 100 ms depolarizing voltage pulse to 0 mV from a holding potential of –50 mV. The I_Ca_-induced SR Ca^2+^ release and the corresponding cytosolic Ca^2+^ rise were simultaneously measured with the current. The amplitude of the I_Ca_ current was normalized to the membrane capacitance termed I_Ca_ density. Single exponential fits were applied to analyze the inactivation of I_CaL_ with tau time constants of inactivation. The magnitude of the Ca^2+^ fluorescence changes were quantified as ΔF/F_0_, where ΔF is the amplitude of the fluorescence transient and F_0_ is the basal fluorescence intensity. I_Ca_ tail current-induced Ca^2+^ transients were measured by repolarization from 100 mV to -50 mV. The Ca^2+^ content of the SR was measured by rapid application of 5 mM caffeine for 1 s. The carried charge was estimated by calculating the integral of caffeine activated I_NCX_. All the experiments were performed at 25–30 °C. The data were analyzed with the Clampfit10.7 (Molecular Devices) and Origin 9 (Origin lab) software.

### 4.9. Statistical Analysis

Statistical comparisons were performed using Student’s t-test or one-way ANOVA followed by the Tukey post hoc test. The statistical significance level was set to *p* ≤ 0.05 (labeled with one asterisk) and *p* ≤ 0.01 (labeled with two asterisks on each plot). Statistical analysis was performed using Origin 9 software (Origin lab) and MS Excel software (Microsoft, Redmond, WA). The data are presented as mean ± SEM.

## 5. Conclusions

A higher SR Ca^2+^ content and more frequent Ca^2+^ sparks underlie the increased arrhythmogenicity of the S4938F-RyR2 mutant. The smaller RyR2 Ca^2+^ leak and suppressed I_Ca_-gated calcium release are likely to result from the close proximity of the mutation and the pore.

## Data Availability

The data presented in this study are available on request from the corresponding author.
